# How to Successfully Choose a Journal

**DOI:** 10.1093/geroni/igaa057.3160

**Published:** 2020-12-16

**Authors:** Laura Sands

**Affiliations:** Virginia Tech, Blacksburg, Virginia, United States

## Abstract

Article submissions to The Gerontological Society of America’s high impact scientific journals continue to increase each year which has led to editors becoming more selective about which articles are accepted for publication. The purpose of this session is to describe how to efficiently and successfully navigate the process of determining which journal is the best fit for your manuscript. Objectives of the workshop include guidance in: (1) understanding the differences in scope and features of each journal; (2) determining which journal is most appropriate for the topic and methods of your manuscript; (3) appreciating how to use journals’ Instructions to Authors to your benefit, and (3) conveying the scientific contribution of your article to the journal.

